# Supplementary material for: Towards Blue AIE/AIEE: Synthesis and Applications in OLEDs of Tetra-/Triphenylethenyl Substituted 9,9-Dimethylacridine Derivatives
Source: Molecules. 2020 Jan 21;25(3):445. doi: 10.3390/molecules25030445 (PMC7037823; doi:10.3390/molecules25030445)
Supplement: Supplementary file 1 [file molecules-25-00445-s001.pdf]

molecules

Supporting Information

Towards blue AIE/AIEE: Synthesis and applications in OLEDs of tetra-/triphenylethenyl substituted 9,9-dimethylacridine derivatives

Monika Cekaviciute [1], Aina Petrauskaite [1], Sohrab Nasiri [1], Jurate Simokaitiene [1], Dmytro Volyniuk [1], Galyna Sych [1], Ruta Budreckiene [2] and Juozas Vidas Grazulevicius [1,*]

[1] Department of Polymer Chemistry and Technology, Kaunas University of Technology, Radvilenu rd. 19, LT-50254, Kaunas, Lithuania
[2] Department of Biochemistry, Lithuanian University of Health Sciences, A. Mickeviciaus st. 9, LT-44307, Kaunas, Lithuania
* Correspondence: juozas.grazulevicius@ktu.lt

1. Methods and Instrumentation

^{1}H NMR and ^{13}C NMR spectra recorded with Bruker Avance III [400 MHz (^{1}H), 100 MHz (^{13}C)] spectrometers at room temperature. All the data are given as chemical shifts δ (ppm) downfield from $Si(CH_3)_4$. Infrared (IR) spectra recorded using PerkinElmer Spectrum GX II FT-IR System. The samples of the solid compounds prepared in the form of KBr pellets. Mass spectra (MS) obtained on the Waters SQ Detector 2. Differential scanning calorimetry (DSC) measurements recorded in nitrogen atmosphere with a Perkin Elmer at DSC 8500 equipment at heating rate of 10 ºC/min. Thermogravimetric analysis (TGA) executed on a Perkin Elmer TGA 4000 apparatus in a nitrogen atmosphere at heating rate of 20°C/min. Melting points measured with Electrothermal MEL-TEMP melting point apparatus. Absorption spectra of dilute (10^{-5} M) solutions in tetrahydrofuran (THF), toluene and films registered on an UV-vis-NIR spectrophotometer Lambda 950 (Perkin-Elmer). Fluorescence spectra, fluorescence quantum yields and fluorescence transients of dilute solutions in THF, toluene (10^{-5} M) or solid films of the compounds were registered with Edinburgh Instruments LS980 spectrometer. Cyclic voltammetry (CV) measurements recorded using a micro-Autolab III (Metrohm Autolab) potentiostat equipped with a standard three-electrode configuration. A three-electrode cell equipped with a glassy carbon-working electrode, an $Ag/AgNO_3$ (0.01 M in anhydrous acetonitrile) reference electrode and a Pt wire counter electrode employed. The measurements carried out in anhydrous dichloromethane with tetrabutylammonium hexafluorophosphate (0.1 M) as supporting electrolyte under nitrogen atmosphere at scan rate of 0.05 V/s. The measurements calibrated using an internal standard, ferrocene/ferrocenium (Fc) system. Oxidation potentials ($E_{1/2}$ vs Fc) for reversible oxidation were collected as average values of the anodic and cathodic peak potentials, Epa and Epc, respectively. Ionization potentials (IP_{EP}) of the vacuum-deposited films of the synthesized compounds were measured by electron photoemission method in air as described before [1]. Hole drift mobilities measured by a time of-flight (TOF) method as reported earlier [2]. The pulsed Nd:YAG laser (EKSPLA NL300, 355 nm, 3-6 ns), Keithley 6517B electrometer, Tektronix TDS 3052C oscilloscope were exploited in the TOF experimental setup. Hole mobility was calculated as $\mu=d^2/U\cdot tt$ using transit time (tt) with the applied bias (U) and the entire thickness (d) of the samples.

Thermo-vacuum deposition technique (Kurt J. Lesker in-built in an MB EcoVap4G glove box) was used for fabrication of electroluminescent devices under pressure lower than 2×10^{-6} mBar. Density-voltage and luminance-voltage characteristics were registered using Keithley 2400C sourcemeter and certificated photodiode PH100-Si-HA-D0 together with the PC-Based Power and Energy Monitor 11S-LINK as it was previously described [3]. External quantum efficiency estimated using the luminance, current density, and EL spectrum. Electroluminescence (EL) spectra recorded by an Aventes AvaSpec-2048XL spectrometer. EL spectra used for calculations of chromaticity coordinates (x, y) of the devices.

1.1. ^{1}H NMR spectra of synthesized compounds

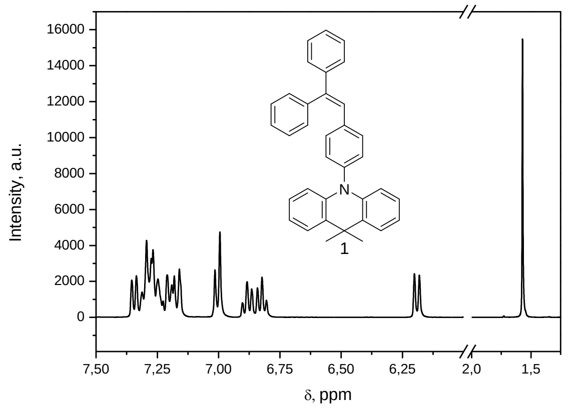

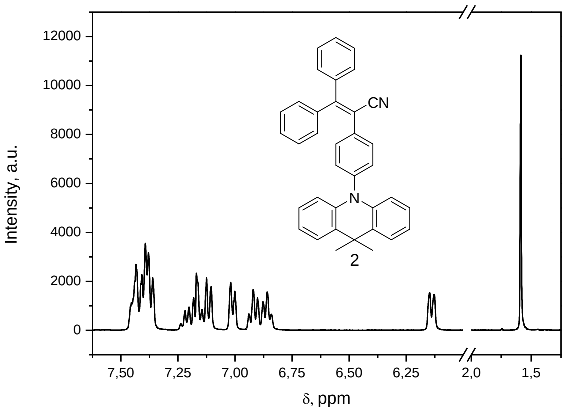

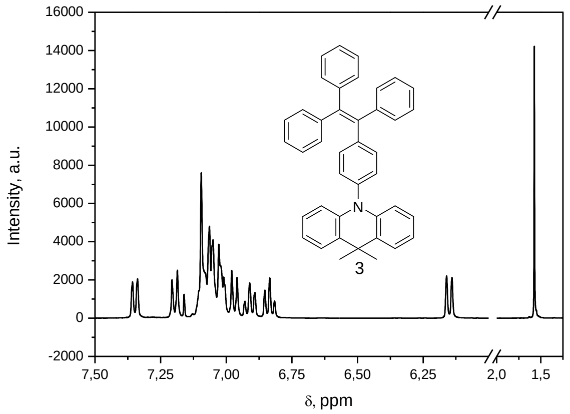

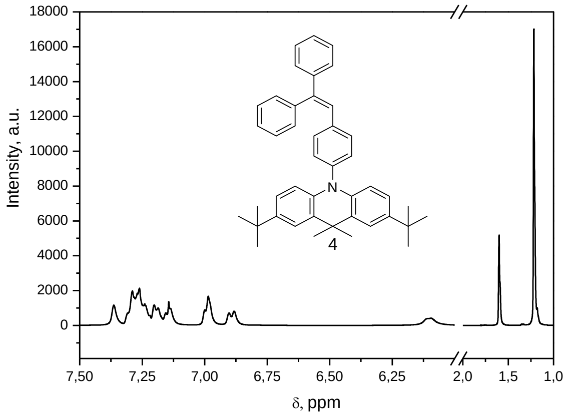

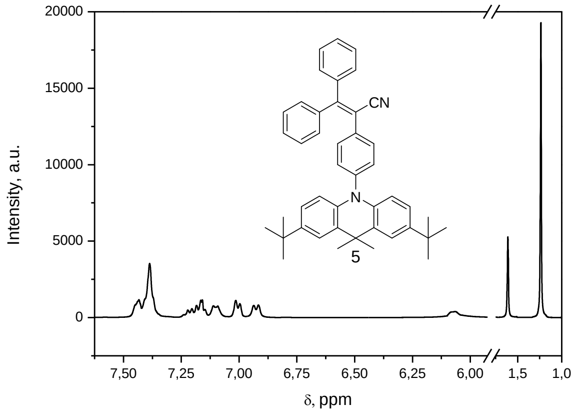

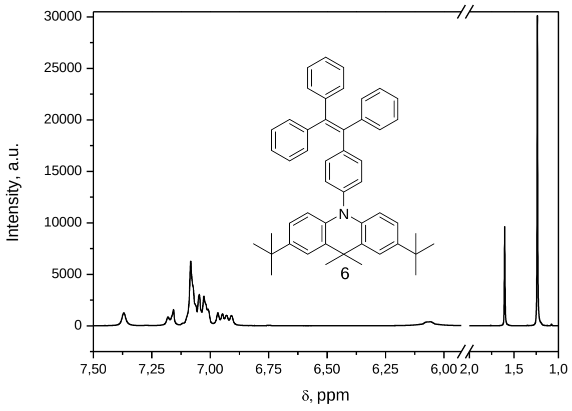

2. Results

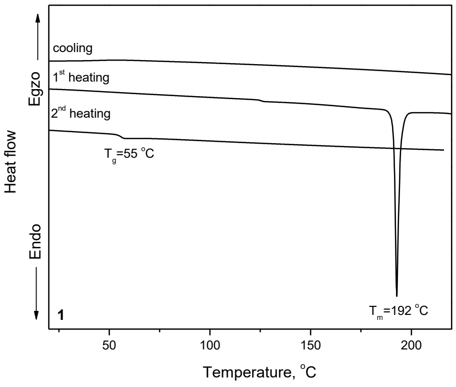

(**a**)

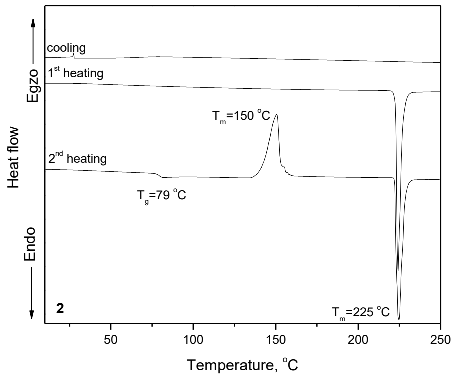

(**b**)

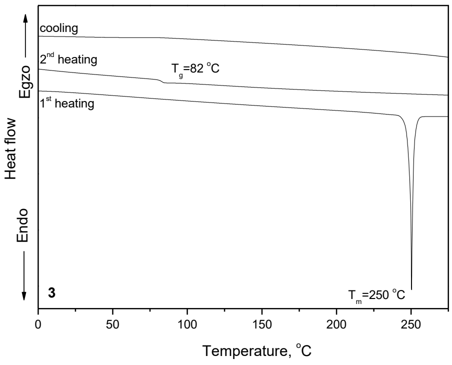

(**c**)

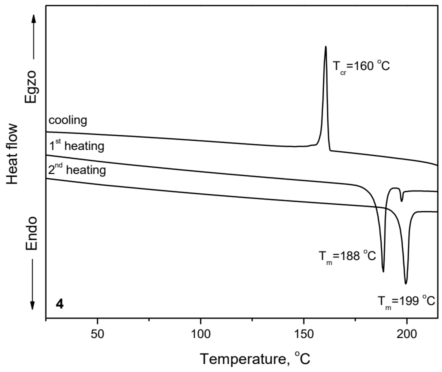

(**d**)

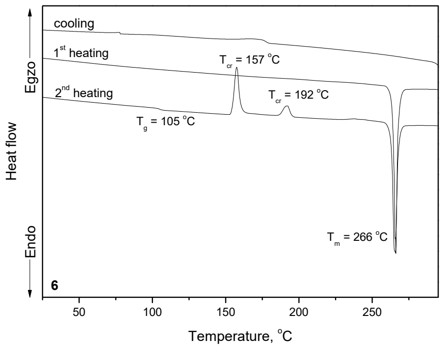

(**e**)

Figure S1. DSC curves for powder of compounds (a) **1**, (b) **2**, (c) **3**, (d) **4** and (e) **6**.

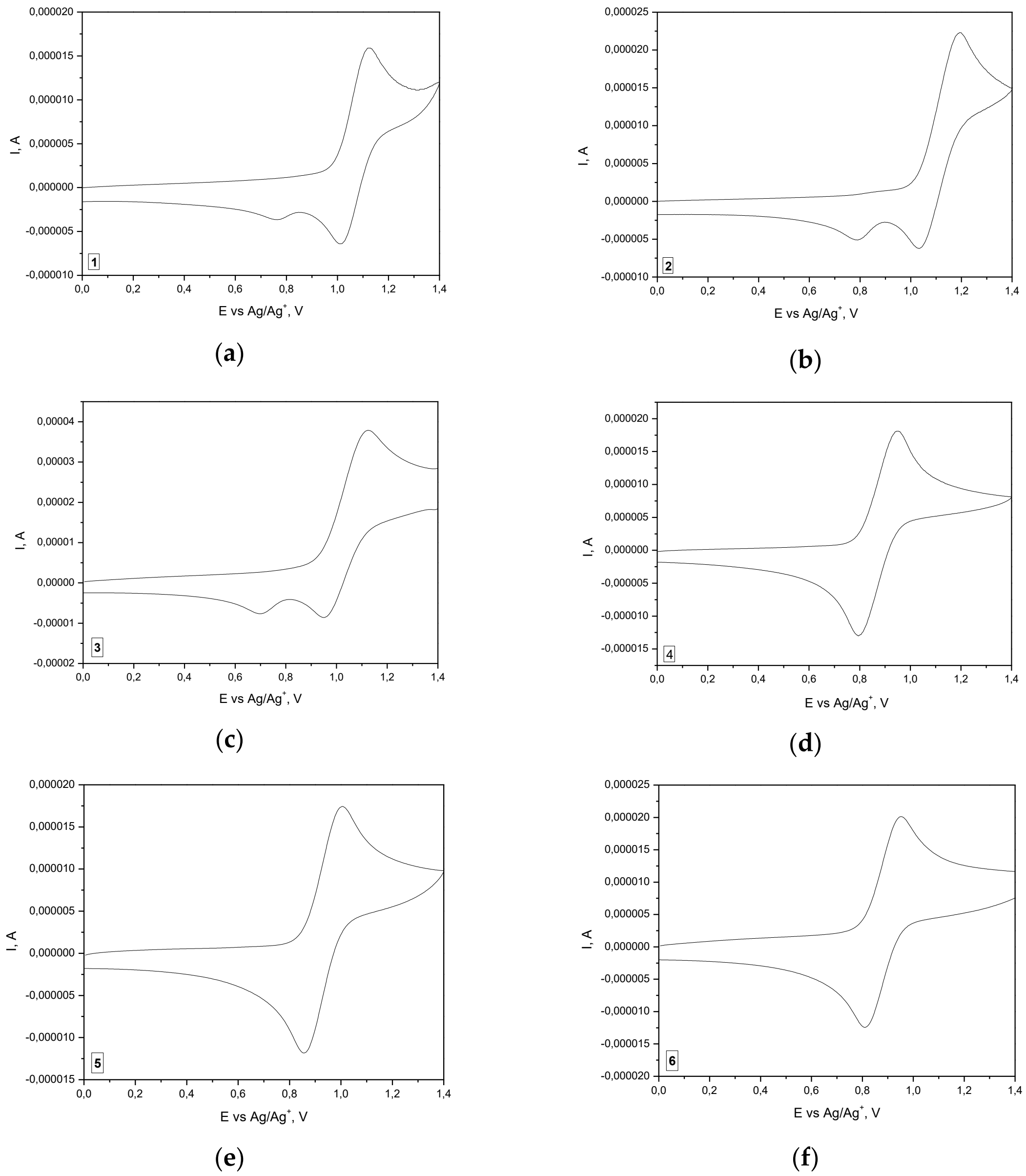

Figure S2. First CV cycle curves of compounds (**a**) **1**, (**b**) **2**, (**c**) **3**, (**d**) **4** (**e**) **5** and (**f**) **6**.

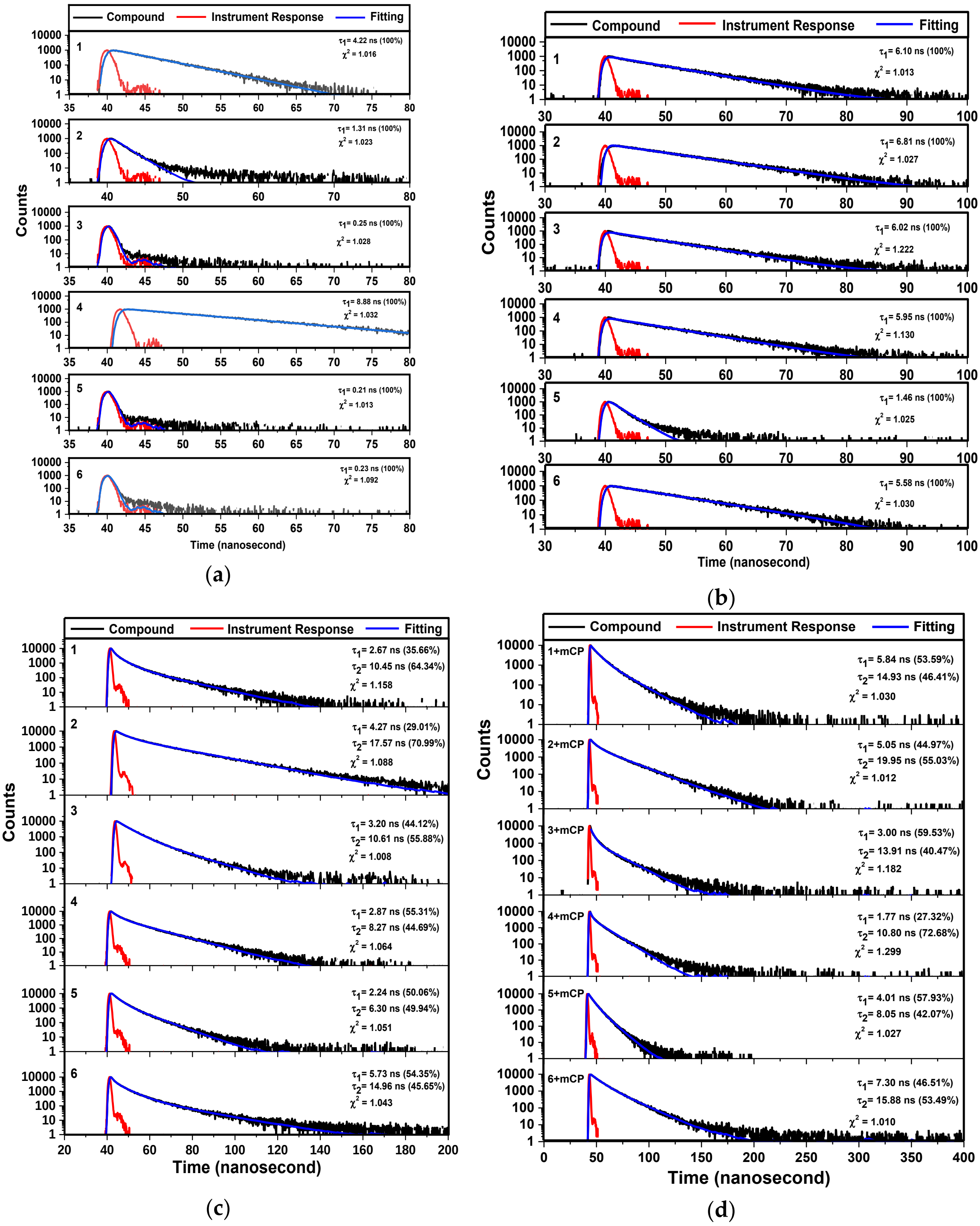

Figure S3. PL decays of compounds **1**–**6** in (**a**) toluene and (**b**) THF solutions and in (**c**) non-doped and (**d**) doped films.

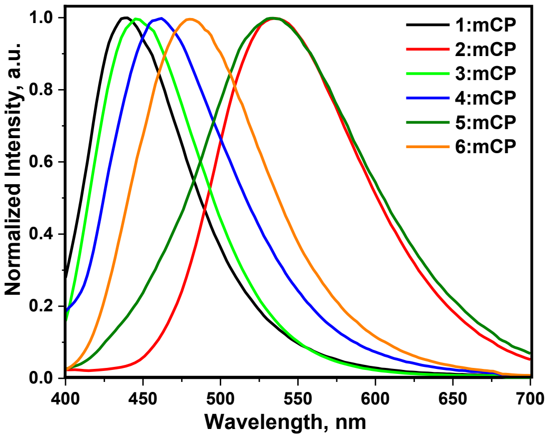

Figure S4. Normalized PL spectra of doped solid films **1–6**:mCP (excitation wavelength was 330 nm).

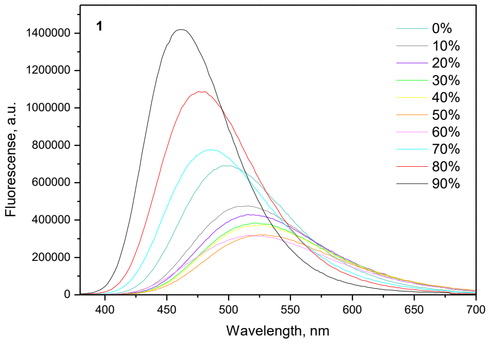

(**a**)

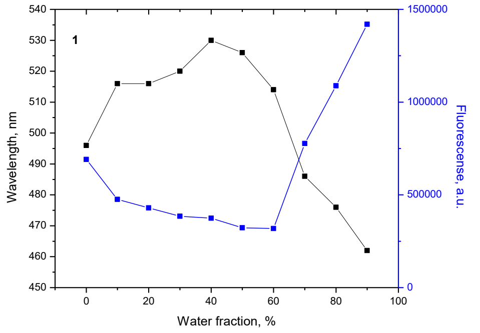

(**b**)

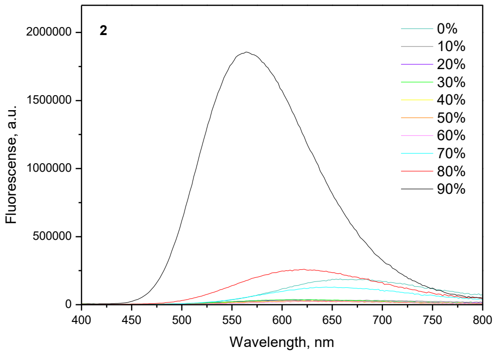

(**c**)

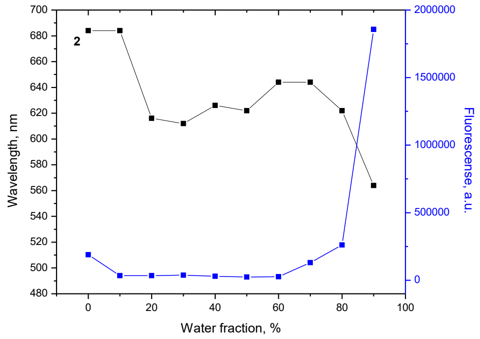

(**d**)

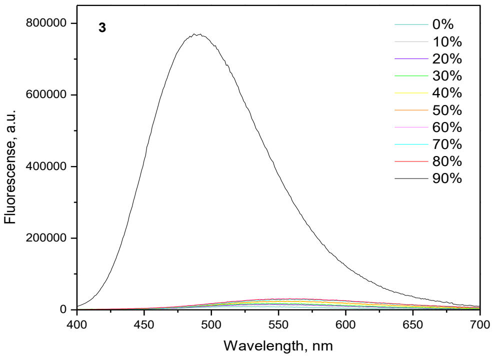

(**e**)

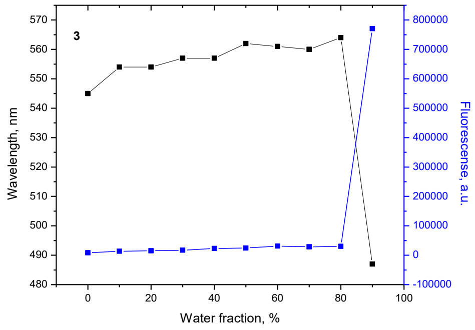

(**f**)

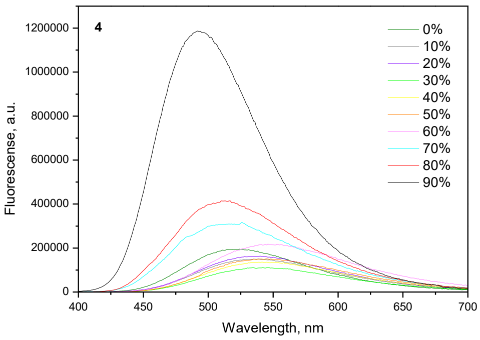

(**g**)

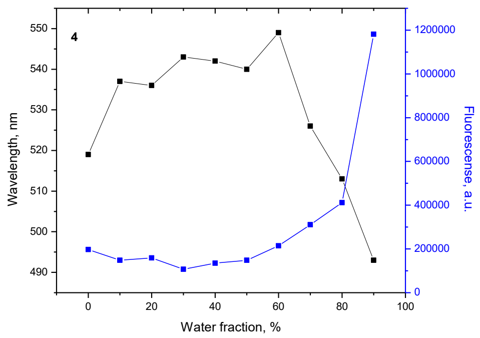

(**h**)

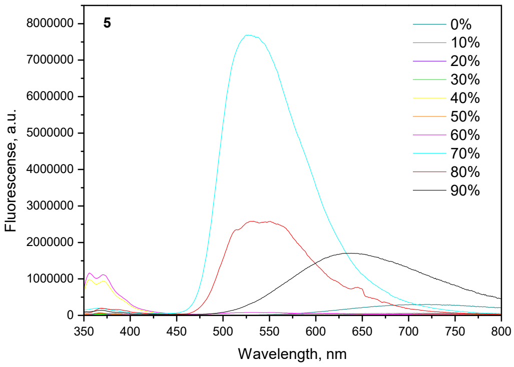

(**i**)

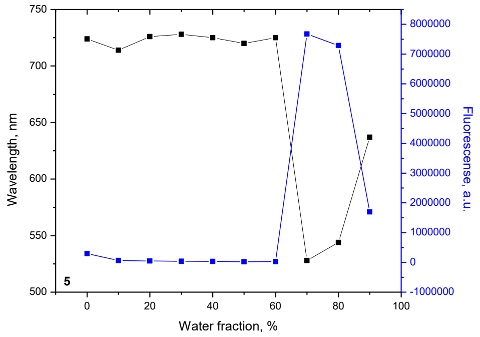

(**j**)

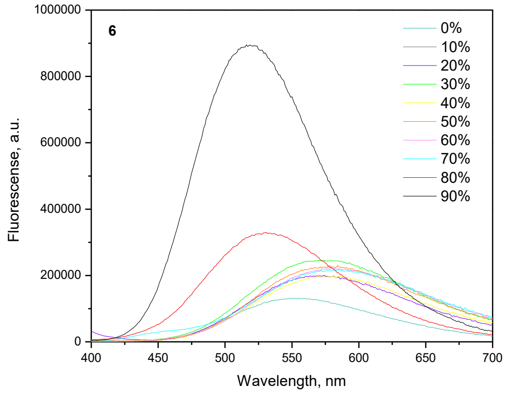

(**k**)

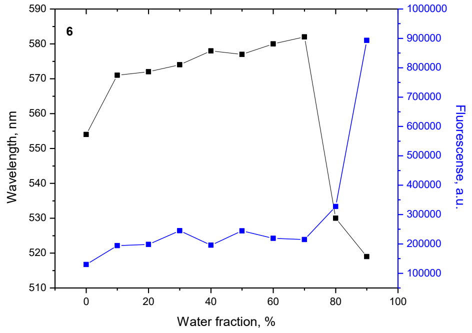

(**l**)

Figure S5. Emission spectra of compounds (**a**) **1**, (**c**) **2**, (**e**) **3**, (**g**) 4 (**i**) **5** and (**k**) **6** in THF/water mixtures (0–90%). Plot of maximum emission intensity and wavelength of compounds (**b**) **1**, (**d**) **2**, (**f**) **3**, (**h**) **4** (**j**) **5** and (**l**) **6** versus water fraction.

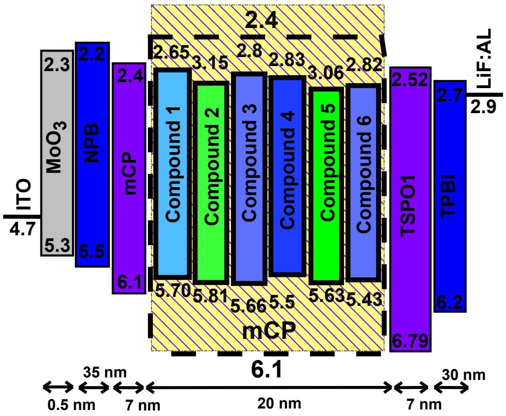

Figure S6. The structure of the non-doped and doped devices. Energy levels are given in eV.

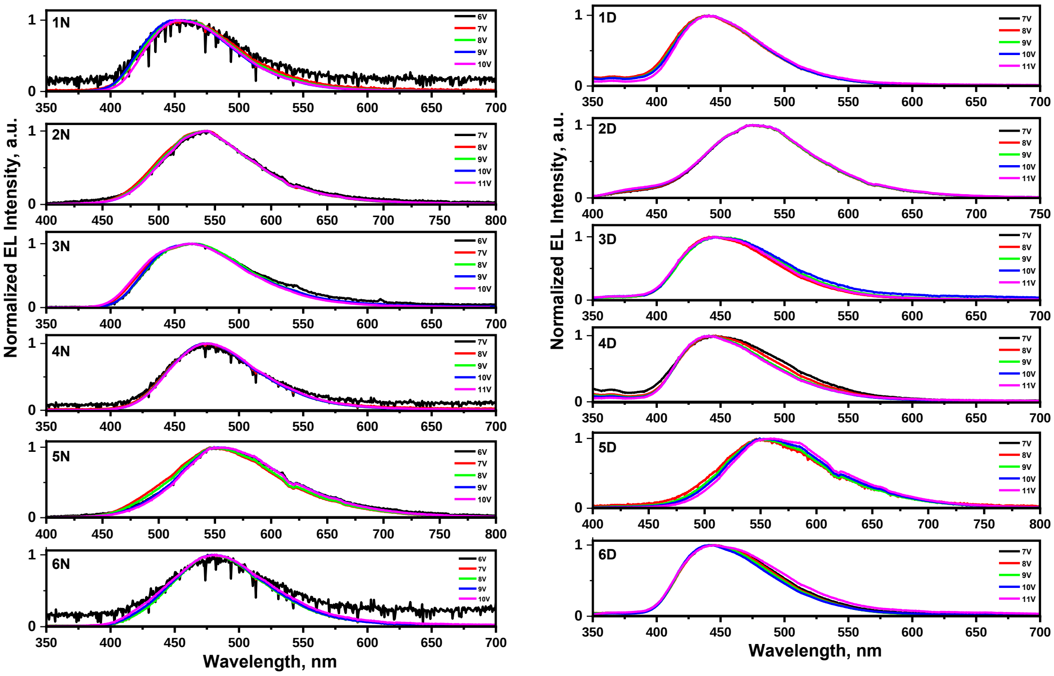

Figure S7. Electroluminescence spectra of fabricated devices at different applied voltages.

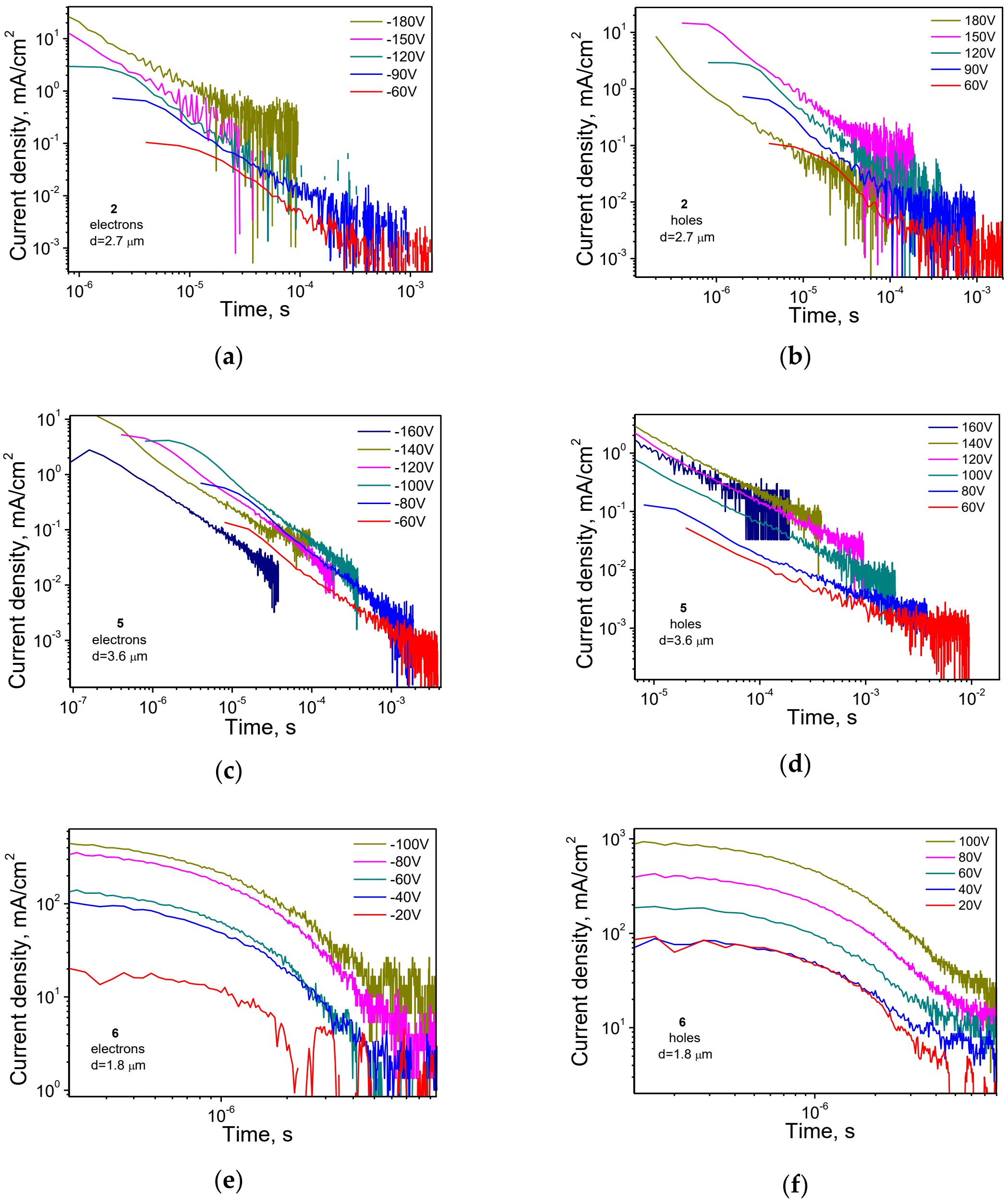

Figure S8. TOF transients of compounds (**a**, **b**) **2**, (**c**, **d**) **5** and (**e**, **d**) **6**.

Table S1. Photophysical parameters for toluene solutions and doped films of compounds 1-6.

**Compound**	**Toluene**				**Doped film**			
	**PLQY, %**	**τ_f (ns)**	**k_r (10^7 s^{-1})**	**k_{nr} (10^7 s^{-1})**	**PLQY, %**	**τ_f (ns)**	**k_r (10^7 s^{-1})**	**k_{nr} (10^7 s^{-1})**
**1**	32	4.22	7.58	16.11	72	9.97	7.23	2.80
**2**	1	1.31	0.76	75.57	88	13.20	6.67	0.906
**3**	<1	0.25	4.00	396.00	71	7.34	9.68	3.95
**4**	39	8.88	4.39	6.87	52	8.26	6.30	5.80
**5**	2	0.21	9.52	466.67	38	5.67	6.71	11
**6**	1	0.23	4.35	430.43	83	11.78	7.05	1.44

The radiative (k_r) and non-radiative (k_{nr}) rate constants using the following equations [4]: $k_r=\frac{PLQY}{\tau_f}$ $k_{nr}=\frac{1}{\tau_f}$ - k_r, where τ_f is average fluorescence lifetime.

Table S2. OLED characteristics for non-doped devices based on nitrogen-containing heteroaromatic carbazole, triphenylamine and acridan moieties substituted by tetraphenylethene and/or triphenylethene units.

Device(Emitter)	V_{on} (V)	λ_{max} (nm)	L_{max} (cd/m^2)	CE_{max} (cd/A)	PE_{max} (lm/W)	EQE_{max} (%)	CIE1931 coordinates (X,Y)	References
**Tetra(tri)phenylethene-substituted acridan derivatives as OLED emitters**								
**1N(1)**	4.4	456	3350	2.23	1.07	1.10	(0.15, 0.13)	This work
**2N(2)**	4.4	540	4940	2.90	1.60	1.59	(0.34, 0.56)	This work
**3N(3)**	5.2	465	2630	2.83	1.78	0.86	(0.16, 0.16)	This work
**4N(4)**	5.4	475	4200	1.10	0.51	0.82	(0.15, 0.22)	This work
**5N(5)**	4.5	550	3360	1.30	0.53	0.45	(0.41, 0.53)	This work
**6N(6)**	4.2	480	4090	1.55	0.89	1.35	(0.17, 0.25)	This work
**Tetra(tri)phenylethene-substituted carbazole/triphenylamine derivatives as OLED emitters**								
**II(1)**	4.4	472	7160	1.9	1.2	1.3	-	[5]
**II(2)**	4.4	492	5130	3.9	2.0	1.7	-	[5]
**I(TPECa)**	4.0	484	7508	3.8	2.7	1.8	-	[5]
**I(TPECaP)**	3.8	488	11060	3.5	2.9	1.7	-	[5]
**I(TTPECaP)**	4.0	488	13650	3.8	2.1	1.8	-	[6]
**A(C4FS1)**	3.8	496	12500	2.8	-	1.08	(0.185,0.38)	[7]
**B(C4FS2)**	3.8	499	3200	0.8	-	0.34	(0.218,0.367)	[7]
**M (p-DPDECZ)**	4.8	524	30210	9.96	5.43	2.73	(0.25,0.52)	[8]
**N (p-DBPDECZ)**	4.2	513	65150	8.60	5.07	3.28	(0.23,0.46)	[8]
**P (m-DPDECZ)**	4.9	489	14980	2.53	0.99	1.26	(0.19,0.30)	[8]
**Q (m-DBPDECZ)**	5.2	500	16410	4.49	2.57	2.16	(0.20,0.34)	[8]
**A (DBPTECZ)**	6.2	513	21054	3.34	-	-	(0.22, 0.41)	[9]
**D1 (1)**	3.81	470	4700	3	2.35	1.62	(0.168,0.209)	[10]
**D2 (2)**	3.99	441	4300	1.4	0.82	1.08	(0.159,0.126)	[10]
**D3 (3)**	3.99	479	2000	3.8	2.6	1.95	(0.174,0.244)	[10]
**D4 (4)**	4.19	499	12100	4.26	2.2	1.6	(0.194,0.463)	[10]
**D5 (5)**	3.79	518	15500	5.8	3	2	(0.248,0.514)	[10]

References

1. Kukhta, N.A.; Volyniuk, D.; Peciulyte, L.; Ostrauskaite, J.; Juska, G.; Grazulevicius, J.V. Structure-property relationships of star-shaped blue-emitting charge-transporting 1,3,5-triphenylbenzene derivatives. *Dyes Pigm* **2015**, *117*, 122–132.
2. Vaezi-Nejad, S.M. Xerographic time of flight experiment for the determination of drift mobility in high resitivity semiconductors. *Int. J. Electron.* **1987**, *62*, 361-384.
3. Greenham, N.; Friend, R.; Bradley, D. Angular dependence of the emission from a conjugated polymer, light-emitting diode: implications for efficiency calculations. *Adv. Mater.* **1994**, *6*, 491–494.
4. Ghosh, S.; Mandal, S,; Banerjee, C.; Govind Rao, V.; Sarkar, N. Photophysics of 3,3′-Diethyloxadicarbocyanine Iodide (DODCI) in Ionic Liquid Micelle and Binary Mixtures of Ionic Liquids: Effect of Confinement and Viscosity on Photoisomerization Rate. *Physical Chemistry B.* **2012**, *116*, 9482.
5. C.Y.K. Chan, J.W. Y. Lam, Z. Zhao, S. Chen, P. Lu, H.H.Y. Sung, H.S. Kwok, Y. Ma, I.D. Williams, B.Z. Tang. J. Aggregation-induced emission, mechanochromism and blue electroluminescence of carbazole and triphenylamine-substituted ethenes. Mater. Chem. C, 2014, 2, 4320-4327.
6. Z. Zhao, C.Y. K. Chan, S. Chen, C. Deng, J.W. Y. Lam, C.K. W. Jim, Y. Hong, P. Lu, Z. Chang, X. Chen, P. Lu, H.S. Kwok, H. Qiu, B.Z. Tang. Using tetraphenylethene and carbazole to create efficient luminophores with aggregation-induced emission, high thermal stability, and good hole-transporting property. J. Mater. Chem., 2012, 22, 4527-4534.

7. G. Sych, J. Simokaitiene, O. Bezvikonnyi, U. Tsiko, D. Volyniuk, D. Gudeika, J.V. Grazulevicius. Exciplex-Enhanced Singlet Emission Efficiency of Nondoped Organic Light Emitting Diodes Based on Derivatives of Tetrafluorophenylcarbazole and Tri/Tetraphenylethylene Exhibiting Aggregation-Induced Emission Enhancement. J. Phys. Chem. C 2018, 122, 14827-14837.
8. H. Shi, D. Xin, X. Gu, P. Zhang, H. Peng, S. Chen, G. Lin, Z. Zhao. B.Z. Tang. The synthesis of novel AIE emitters with the triphenylethene-carbazole skeleton and para-/ meta-substituted arylboron groups and their application in efficient non-doped OLEDs. J. Mater. Chem. C, 2016, 4, 1228-1237.
9. H. Shi, X. Zhang, C. Gui, S. Wang, L. Fang, Z. Zhao, S. Chen, B.Z. Tang. Synthesis, aggregation-induced emission and electroluminescence properties of three new phenylethylene derivatives comprising carbazole and (dimesitylboranyl)phenyl groups. J. Mater. Chem. C, 2017, 5, 11741-11750
10. S. Nasiri, M. Cekaviciute, J. Simokaitiene, A. Petrauskaite, D. Volyniuk, V. Andruleviciene, O. Bezvikonnyi, J.V. Grazulevicius. Carbazole derivatives containing one or two tetra-/triphenylethenyl units as efficient hole-transporting OLED emitters. Dyes and Pigments, 2019, 168, 93–102.
